# Collective Domain Motion Facilitates Water Transport in SGLT1

**DOI:** 10.3390/ijms241310528

**Published:** 2023-06-23

**Authors:** Marko Sever, Franci Merzel

**Affiliations:** 1 Theory Departnemt, National Institute of Chemistry, Hajdrihova 19, 1000 Ljubljana, Slovenia; marko.sever@ki.si; 2 Faculty of Pharmacy, University of Ljubljana, Aškerčeva 7, 1000 Ljubljana, Slovenia

**Keywords:** molecular dynamics simulations, SGLT1 protein, transmembrane water transport, diffusion, intrinsic domain motion, principal component analysis

## Abstract

The human sodium–glucose cotransporter protein (SGLT1) is an important representative of the sodium solute symporters belonging to the secondary active transporters that are critical to the homeostasis of sugar, sodium, and water in the cell. The underlying transport mechanism of SGLT1 is based on switching between inward- and outward-facing conformations, known as the alternating access model, which is crucial for substrate transport, and has also been postulated for water permeation. However, the nature of water transport remains unclear and is disputed along the passive and active transport, with the latter postulating the presence of the pumping effect. To better examine the water transport in SGLT1, we performed a series of equilibrium all-atom molecular dynamics simulations, totaling over 6 μs of sample representative conformational states of SGLT1 and its complexes, with the natural substrates, ions, and inhibitors. In addition to elucidating the basic physical factors influencing water permeation, such as channel openings and energetics, we focus on dynamic flexibility and its relationship with domain motion. Our results clearly demonstrate a dependence of instantaneous water flux on the channel opening and local water diffusion in the channel, strongly supporting the existence of a passive water transport in SGLT1. In addition, a strong correlation found between the local water diffusion and protein domain motion, resembling the “rocking-bundle” motion, reveals its facilitating role in the water transport.

## 1. Introduction

SGLT1 is a membrane cotransporter that is essential for glucose, galactose, and water uptake from the intestine [1,2]. Its functional role of co-transporting substrates across the membrane is associated with highly coordinated structural transitions between its corresponding conformational states, which occur generally in an active manner. SGLT1 belongs to the membrane protein family of sodium solute symporters, sharing a common structural core motif called ‘LeuT-fold’ and playing a crucial role in the physiology of the intestines, brain, kidney, thyroid, and skin, representing an important target for therapeutic intervention in the treatment of diabetes, diarrhea, depression, obesity, etc. [3,4]. The two major conformations that the SGLT1 cotransporter occupies during its working cycle are the inward-facing (IF) and outward-facing (OF) states [5]. This functional mode of the transportation of substrates via cotransporters is known as the alternating-access mechanism [6]. The mechanism has received substantial support from structural, kinetic, and biochemical studies [7,8,9]. It relies on a high level of coordination between the cytoplasmic and the extracellular gating mechanisms so that the substrate-binding sites are exposed only to one side of the membrane at a time [10]. In addition to its capacity to transport substrates, the SGLT1 protein also facilitates the movement of water across the cell membrane. It has been postulated that the water transport in cotransporters could be attributed to two fundamentally different mechanisms: passive osmosis-driven transport [11] and active cotransport-driven water transport [12].

A recent study [13] on water permeation through SGLTs suggests that water permeates through the sugar transport pathway, which provides a major pathway for passive water flow across the small intestine. Additional experimental evidence in favor of the passive water transport in SGLT1 was provided by Erokhova et al. [14], who found that approximately $10^{9}$ water molecules are transported per protein per second, which significantly exceeds the number of a few hundred water molecules that could be expected from the “pumping effect” during the substrate’s molecule turnover. These results suggest a strong influence of SGLTs in regulating the transmembrane water flux. Molecular dynamics (MD) simulations of the bacterial homolog vSGLT confirmed the passive water transport capability in its inward-facing state [15,16]. Extensive MD simulations carried out on a variety of transporter superfamilies concluded that water-conducting states likely represent a universal phenomenon in membrane transporters [17], and that this phenomenon might be a consequence of transporter reliance on large-scale motions. However, it is still unknown how all representative states in the transport cycle are permeable to water and how the inherent domain motion affects the characteristics of water permeability. Namely, an enhanced water transport in SGLT1 can occur in the form of a pulse in a given state or transition between states, or is present as constant leakage during the overall gating motion. Thus, it appears necessary to examine the protein dynamics and obtain insight into the mechanistic details of water permeation, which could be best visualized and understood through MD simulations. Transport in membrane proteins involves highly diverse events of structural changes, ranging from local rearrangements at the binding sites and their gating elements, to global conformational transitions. This transport process is not trivial to describe since the protein atoms are in constant thermal motion and, furthermore, the gates of proteins are opening and closing. Numerous pathways for water and substrate molecules are transient and only certain fluctuations or rearrangements in the protein structures make the further travel of molecules possible. Depending on the timescale of these events, their investigation may be covered with conventional MD simulations or require different model simplification methods or sampling enhancements.

In this study, we developed a unique approach to quantify the dynamic features of the protein and correlate them with the capacity of the protein to permeate water.

## 2. Results

To elucidate the underlying molecular mechanism of water transport in SGLT1, we performed a large set of extended equilibrium MD simulations in the homology-modeled SGLT1 systems developed in [18], obtained from Prof. Grabe via personal communication. In particular, we simulated SGLT1 systems in outward-facing (OF) conformations: (a) a plain apo form with no ligands and no preset bound sodium ions (p-OF), (b) a plain apo form with two preset sodium ions (s-OF), and (c) a phlorizin-bound complex (phl-OF), and two SGLT1 systems in the inward facing (IF) conformation, (d) a plain apo form with no ligands(p-IF), and (e) a complex with the substrate molecule galactose bound (gal-IF). Explicit dynamic representations of these molecular species using all-atom MD simulations enabled us to closely probe many possible factors by which the transport function of SGLT1 can be affected. Each system was inserted into a 3:1 POPC:CHOL bilayer, solvated by a 0.15 M NaCl aqueous solution, as described in Section 4, and simulated several hundreds of nanoseconds under constant pressure and temperature, with applied periodic boundary conditions (PBC).

**Figure 1:**
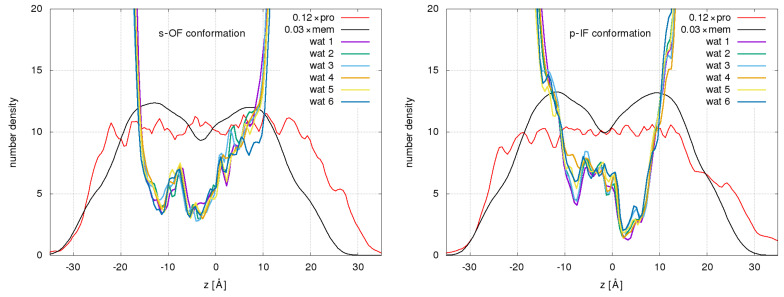
Water density profiles relative to the scaled protein (red curve) and membrane profiles (black) are shown as functions of the *z* coordinate along the membrane normal for the preset sodium-bound OF system and plain IF system, respectively, during sequential 100 ns sections of individual MD trajectories. z=0 corresponds to the *z* coordinate of the center of mass (CoM) of the protein.

The density profiles of water, protein, and membranes for the s-OF and p-IF systems, respectively, are presented in Figure 1. U-curved profiles standing for the distribution of water molecules are clearly biased and demonstrate asymmetry either toward the extracellular side on the right (OF) or toward the intracellular side on the left in the case of the IF system. Interestingly, the minimum water profile, which is located around the sugar-binding site in the IF conformation, shifts by around 10 Å when switching between IF and OF. The minima, most likely indicating constriction points of permeation pathways, are lower in the cases of IF than in the OF profiles. Water profiles in both systems exhibit significant fluctuations. The other systems, p-OF, phl-OF, and gal-IF, share all the above-mentioned features specific to OF and IF systems.

Our first task is to identify water molecule permeation events in simulated systems. The permeation event is defined as a complete passage of a water molecule through the protein in both directions, from one side of a membrane to the other, and is calculated as described in Section 4.

**Figure 2:**
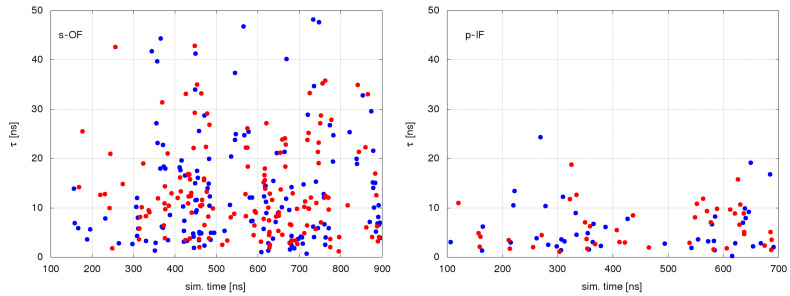
The duration and occurrence of water permeation events throughout the simulation in the s-OF and p-IF systems: red dots indicate efflux and blue dots indicate the influx of water molecules.

In all simulated systems, we found a significant number of water permeation events. Figure 2 shows water permeation events in two systems, s-OF and p-IF, as functions of the simulation times. Dots indicate the occurrences of water permeation in one or the other direction. Ordinate values of individual dots indicate the duration of permeation through the protein. There is no indication of biased water flux through the protein in either direction. Given the absence of the osmotic gradient, such a result reveals the passive mode of water transport. What is notable is the difference in the water transport intensity between the two systems, with an enhanced transport in s-OF relative to the p-IF system.

**Figure 3:**
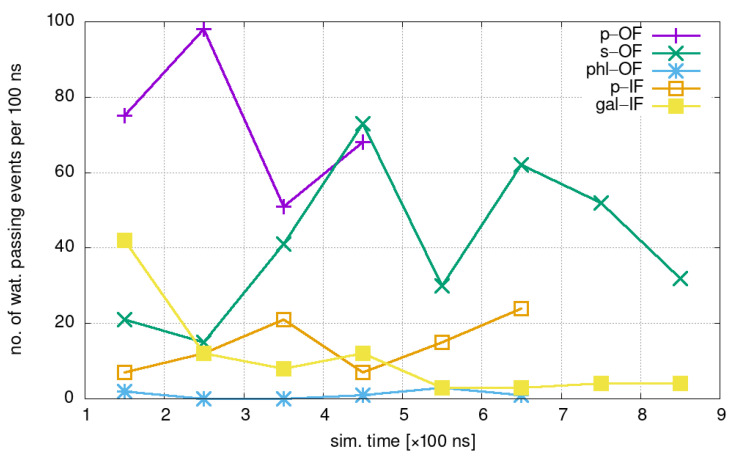
Water passes during consecutive 100 ns intervals of MD simulations of different conformational states.

Our main observable is the number of water molecule permeation events per unit time, $n_{w}$, including the water transport in both directions. The net amount of water permeation events per 100 ns in simulated systems involving different states of the protein are shown in Figure 3.

The most abundant water transport is found in the p-OF state while the weakest is with the bound inhibitor (phl-OF), which demonstrates the expected strong inhibiting effect on the water transport of phlorizin.

$n_{w}$ is directly related to the diffusion permeability, $p_{d}$, of the membrane channel, which quantifies the exchange of water molecules between the two compartments at equilibrium (see Methods). Another characteristic quantity accounting for transport through the water channel is osmotic permeability, $p_{f}$, relating the water flux to osmotic pressure difference. According to the collective diffusion model of Zhu et al. [19], $p_{f}$ can be obtained from an equilibrium MD simulation in the absence of an osmotic gradient, by evaluating the one-dimensional diffusion of the collective variable quantifying the net amount of water permeation. We calculated $p_{f}$ from the mean square displacement of the full permeation events. The results for s-OF and the p-IF system are summarized in Table 1. The calculated permeabilities are within the range of experimentally derived values [14]. It has been shown that when water molecules permeate in the form of a single-file water channel, the ratio $p_{f}/p_{d}$ is constant and equal to N+1, where *N* is the number of water molecules always present and is aligned in one row of the single-file channel [20]. Small values below 2 of the $p_{f}/p_{d}$ ratio given in the last column of Table 1 indicate that it is unlikely that the water transport in SGLT1 is a single-file transport.

In the absence of osmotic and hydrostatic pressure, the frequency of permeation events per unit time is an intrinsic property of the water channel, and should depend on the protein structure-dependent physicochemical factors of the channel.

**Figure 4:**
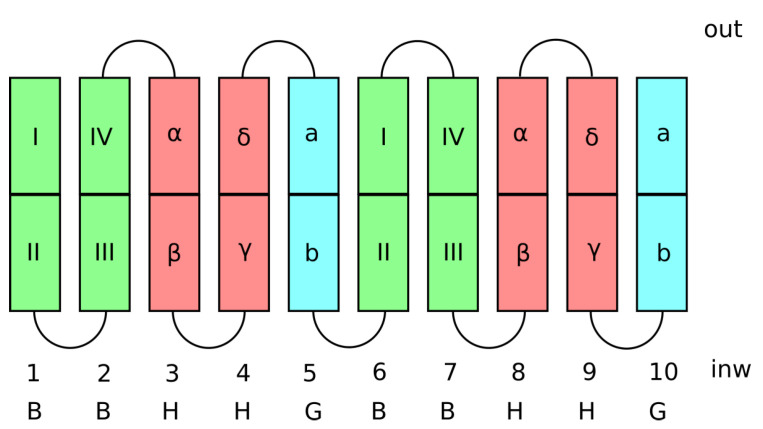
Schematics of the TM helices in the SGLT1 forming bundle (green), hash (red), and gate (cyan) domains.

The most representative structural features of SGLT1 are the 10 transmembrane helices (TM1–TM10) arranged in 2 inverted repeats (TM1-5 and TM6-10). In terms of domain organization, there are two primary domains consisting of transmembrane segments TM1, 2, 6, and 7, forming the core or the so-called “bundle domain”, while TM3, 4, 8, and 9 form the scaffold or “hash domain”. Both primary domains are linked by helices TM5 and TM10, forming the “gating domain” [21]. The organization of the TM helices in SGLT1 is schematically shown in Figure 4.

**Figure 5:**
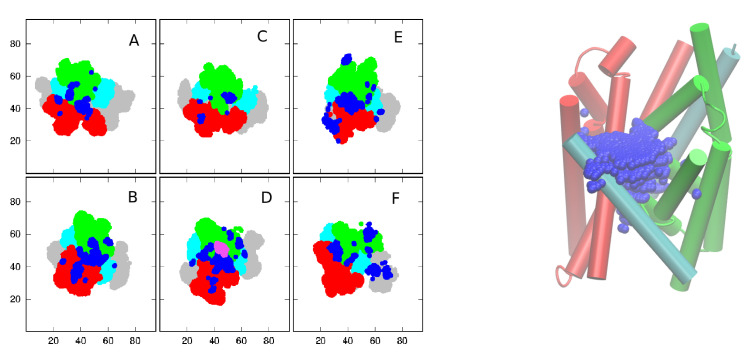
Left: water distribution (dark blue) within SGLT1 (phl-OF conformation) with marked hash (red), bundle (green), and gate (cyan) domains in consecutive layers: (**A**) [−15, −10], (**B**) [−10, −5], (**C**) [−5, 0], (**D**) [0, 5], (**E**) [5, 10], and (**F**) [10, 15] (all *z* values in Å). The phlorizin molecule is marked in pink. Right: 3D representation.

It is important to know how water is distributed within the protein, in particular relative to the bundle, hash, and gate domains. The 2D slices of atom density averaged over 100 ns of the MD simulation of the phl-OF system, and belonging to different atom groups in consecutive 5 Å thick layers along the *z*-axis (perpendicular to the membrane), are shown in Figure 5. They reveal that water is not confined to a single channel but is distributed over different locations spread mainly between bundle and hash domains. All water within the protein is taken into account. As expected for OF systems, the constriction regions for water are located on the intracellular side z<0 Å. The phlorizin molecule is located in its binding site 0<z<5 Å, colored pink in the panel Figure 5D.

The relatively broad distribution of water does not support a single-water transport in SGLT1.

The rate-determining factors of water permeation should be sought in a way to investigate how the water within the channel interacts with the protein. We focus only on water molecules taking part in full permeation events, i.e., which pass the entire membrane span (Δz=30 Å).

**Figure 6:**
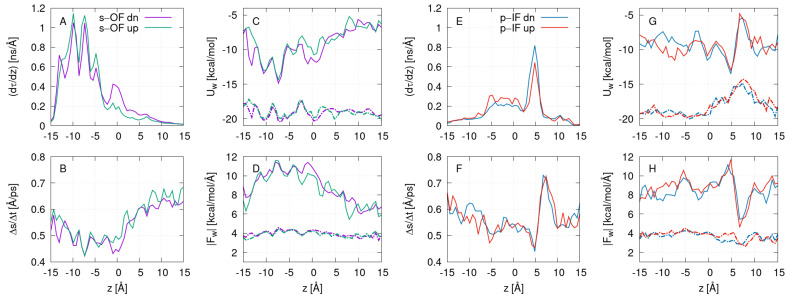
Interaction between related properties of water molecules that have successfully traversed the channel, for the s-OF ((**A**–**D**) panels, green and violet curves) and for p-IF ((**E**–**H**), red and blue curves) system, respectively, as a function of *z*. Each passing direction is treated separately. (**A**,**E**) show the distribution functions of the residence time per unit length for individual water molecules; (**B**,**F**) display the local water molecule mobilities; (**C**,**G**) show water molecule interactions with protein only (full lines) and total environment (dashed lines); (**D**,**H**) show the average magnitudes of the force acting on individual molecules due to protein (full lines) and the total environment (dashed line). All quantities are averaged over the last 100 ns of MD runs.

Panels A and E in Figure 6 show the distribution functions of the single water molecule’s residence time per unit length. Regions of longer residence times agree with the location constriction parts of the channel in each conformation: on the left side (z<0) for the s-OF system (A) and on the right side (z<0) for the p-IF system (E). Note that the integral $\int_{-\Delta z/2}^{\Delta z/2}\left(d\tau /dz\right)dz$ equals the time that one water molecule on average needs to pass the channel. Distributions for upward- and downward-moving molecules are similar to each other, suggesting the same pathways of water permeation in both directions. Water mobilities shown in Figure 6B,F, which are calculated as average displacements that single water molecules perform during 2 ps timesteps, are reduced in constriction regions, except for the range 5<z<10 Å in the p-IF system, which is found to be related to the hydrophobic character of the channel due to the presence of hydrophobic residues F101, F453, and L87. It turns out that the water mobility patterns align well with the profiles of water molecule interaction potential energy with the protein, as depicted in Figure 6C,G (full lines), and not with the net interaction potential energy, taking into account the total environment, including the solvent (dashed lines). The hydrophobic character exposed in the p-IF system is seen as a peak at z=7.5 Å. The total interaction potential for s-OF is relatively flat at a constant value of −20 kcal/mol, apart from the modest variations of magnitude ±1 kcal/mol in the range of z<0 Å. There is an evident barrier at the height of ≈ 4 kcal/mol with a peak matching the hydrophobic part of the channel in the p-IF system. We also evaluated the magnitude of forces, $|\Delta U_{w}/\Delta s|$, acting on a single water molecule due to the total environment, and separated the contribution only from the water–protein interactions. The profiles of forces due to protein interactions in Figure 6D,H show inverted characters to water mobility graphs, while the total force distributions demonstrate relatively flat behavior.

**Figure 7:**
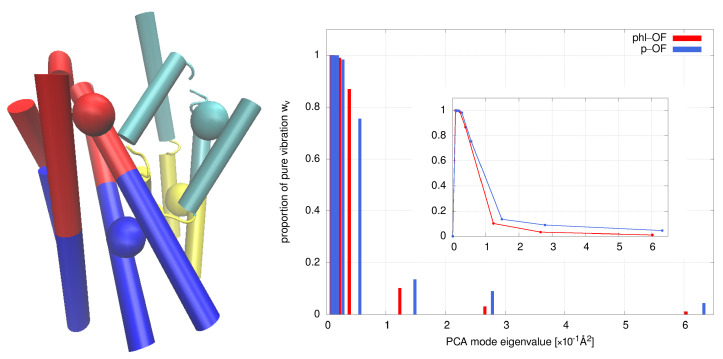
Four-bead model of SGLT1 representing the bundle and hash domain (**left**) and distribution of PCA eigenvalues for p-OF and phl-OF (**right**); the inset presents connected distributions.

The next physical factor we examine, which is expected to influence the water transport, is the internal protein dynamic. A commonly used tool for analyzing protein motions obtained from MD simulations is the principal component analysis (PCA) [22]. Slow- or low-frequency PCA modes are often associated with the functional modes of various protein systems [23,24,25] and are promising for elucidating transport mechanisms. To focus on the relative motion of the domain while minimizing the number of degrees of freedom, we define a four-bead model of SGLT1, two beads for the hash domain and two for the bundle domain, as shown in the left half of Figure 7, where one bead represents the center of mass of the lower half and the other the center of mass of the upper (extracellular) half of each domain. In this way, we can easily capture the rigid body motion, which is expected to be present for an inverted repeat structure of SGLT1, given that specific types of motion, such as “rocking bundle”, are commonly postulated in proteins with the LeuT fold [26].

PCA eigenvalues can be used to assess protein softness (see Methods), which is based on a relationship between structural flexibility and dynamic fluctuations.

As an example, protein softness σ, defined by Equation (6), is calculated from the four-bead PCA analysis from over the last 100 ns section of MD trajectories of phl-OF and p-OF, which were found to differ in their ability to permeate water according to the results in Figure 3. We obtained σ(p − OF) = 0.027 Å${}^{2}$ and σ(p − OF) = 0.011 Å${}^{2}$, indicating large differences in the presence of dynamic fluctuations in both systems. PCA eigenvalues with corresponding vibrational weights $w_{v}$ are shown on the right half of Figure 7.

In addition to dynamic fluctuations of the transporter, one would intuitively expect the opening of the channel as the main factor for water transport. However, defining the channel opening in SGLT1 is not straightforward. Here, we define a measure of the channel opening $d_{\min}$ as the minimum in the number density of the water profile obtained for a given time interval, as shown in Figure 1.

**Figure 8:**
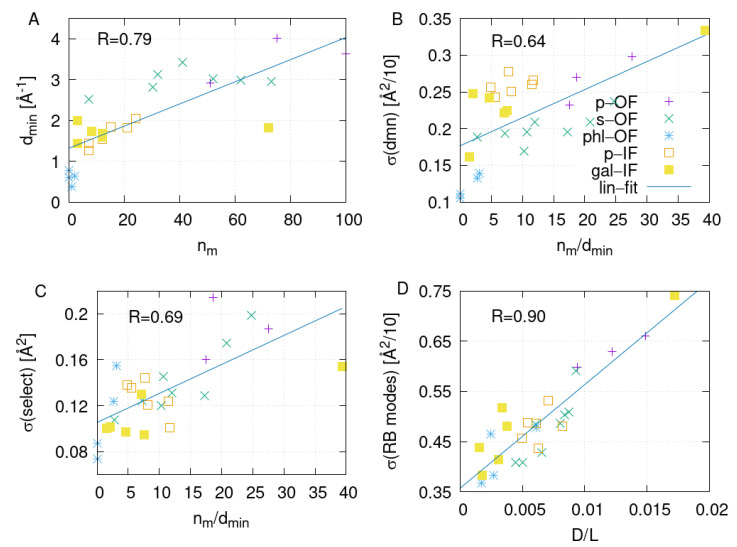
Correlation graphs: (**A**): number of permeation events per unit time, $n_{w}$, versus channel opening, (**B**): ratio $n_{w}/d_{\min}$ versus softness of domain fluctuations, (**C**): $n_{w}/d_{\min}$ versus softness of the selected channel residue fluctuations and (**D**): local water diffusion in the constriction range D/L versus the softness of the rocking bundle type of domain motion.

In order to check the impact of the channel opening to water permeation in SGLT1, we correlate the number of permeation events per unit time, $n_{w}$, with $d_{\min}$, calculated for 100 ns sections for all simulated systems in OF and IF conformations. The correlation is shown in Figure 8A. The channel opening obviously correlates well with the water flux; however, it cannot be considered the only rate-determining factor for water permeation, which can be argued by looking at a relatively large dispersion in $n_{w}$ at fixed values of opening parameter $d_{\min}$ in Figure 8A. In order to check the relationship between the remaining dependence of the water permeation on protein dynamics, we calculate the correlation between the quantity $n_{w}/d_{\min}$ and the softness of the domain fluctuations $\sigma_{dmn}$ obtained from the PCAs of the four-bead model for all 100 ns sections of the simulated systems. In addition, we performed the PCA analysis on select (32) residues along the water pathway that were in contact with water in all simulated systems and calculated $\sigma_{sel}$ for all 100 ns sections. Results that demonstrate good correlation in both cases are shown in Figure 8B,C.

**Figure 9:**
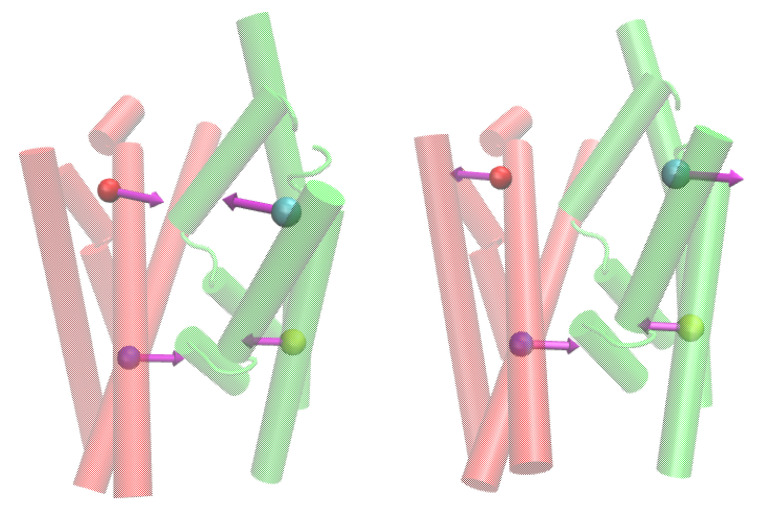
Water flux facilitating the type of rigid domain motion (the bundle domain is colored green, the hash domain is red).

Given that the channel permeability, in the case of the passive transport, equals the product of the channel’s cross-section and local diffusion, according to Equation (2), we recognize that the ratio $n_{w}/d_{\min}$, which scales as p/S, should be proportional to the local diffusion constant of water over the channel length, D/L. Thus, we correlate local water diffusion D/L, which we calculate from the mean square displacements of water molecules in the constriction region of thickness 5 Å for each system, with the softness of a given predefined mode, as defined in Equation (7). We observe a significant correlation if we correlate local water diffusion with the two modes associated with the domain motion (Figure 8D), which affects the distance between the bundle and hash domain, as depicted in Figure 9. Such a finding is meaningful, as water pathways are located on the interface between the bundle and hash domains. These results clearly show that the presence of thermal fluctuations in the SGLT1 systems resembling the so-called rocking-bundle domain motion types, significantly affect water transport.

## 3. Discussion

The ability of the SGLT1 to provide the transmembrane cotransport of water should be rooted in its structural plasticity, i.e., in its sampling of many conformationally distinct states. This factor becomes particularly important in the case of water molecule movement through narrow protein channels, as the magnitude of channel structural fluctuations, due to the thermal motion, may significantly affect the strength of the interaction between water and the channel. Moreover, an increased flexibility of the protein allows for larger displacements in response to the perturbing influence of water molecules in the channel. Our results meaningfully corroborate the importance of the channel’s flexibility in its transporting function, which is in line with previous works, indicating the dynamic flexibility of the protein channel as the origin of function and selectivity [27]. In addition, it was recently shown that externally triggered enhanced structural fluctuations in the glucose transporter (GLUT1) are responsible for the increased permeation of glucose and water [28]. In this context, one needs to capture conformational fluctuations of the transporter, establish a link to the formation of water permeation pathways, and describe the dynamics of water molecules as consequences of structural features.

Obtaining a physically meaningful picture of water transport allows us to rationally modulate the activity of these transporters by designing proper pharmacological means.

## 4. Methods and Materials

### 4.1. Homology Models

Homology models of the inward-facing and outward-facing conformations of SGLT1 were kindly provided by M. Grabe/P. Bisignano from the Cardiovascular Research Institute, University of California; the models were provided as a result of their study [18]. They constructed the IF conformation of the homology model based on the X-ray structure of the sodium–glucose transporter from Vibrio parahaemolyticus (vSGLT) [29,30]. The OF conformation was based on the TM1–TM10 helices from the X-ray structure of the N-acetylneuraminic acid transporter from Proteus mirabilis (SiaT), while loops were modeled by using vSGLT as the template [31]. The outward-facing model also included the TM0 (also called TM-1) helix, which does not represent a part of the core structure of the transporter, which are TM1–TM10 [1]. Helices from TM1–TM10 also form part of the functional domain structure of SGLT1.

### 4.2. Docking of Phlorizin

The docking calculation of the inhibitor phlorizin was performed with the BIOVIA Discovery Studio software suite [32]. The OF conformation was used, as the literature shows that the inhibitor molecule binds from the extracellular side to the OF conformation [18]. The model of the OF conformation of SGLT1 was prepared in a form suitable for docking with the use of the “Protein Prepare” protocol of BIOVIA Discovery Studio. The protonation state of the cotransporter was set to pH 7 with the use of PROPKA [33]. The structure of phlorizin was obtained from the PubChem [34] database in .sdf format and was visually inspected regarding the correct atom connectivity. We optimized the geometry of the inhibitor using a Gaussian program [35] with the 6–31 g* basis set, and the molecule was subjected to energy minimization to generate conformers. A spherical docking area with a radius of 15 Å was chosen, which was centered in the area of residues, which are—by mutagenesis studies—confirmed to participate in the binding of phlorizin [18]. The GOLD [36] docking algorithm, with its default GoldScore [37] scoring function, was used. From the top 100 obtained poses, the top 10 poses were visually inspected. The final pose was chosen based on the results of the scoring function, visual inspection of the poses, and chemical intuition.

Our docking results are in line with previous work, as referenced in the literature; our final docking pose corroborates with the mutagenesis studies referenced in the literature [18]. The sugar moiety of the inhibitor places itself into the sugar-binding site in the SGLT1 cotransporter, while the aglycone tail of the inhibitor is placed in the extracellular vestibule.

### 4.3. MD Simulations

Explicit solvent atomistic simulation systems were prepared using the online server CHARMM-GUI [38]. The orientation of the transporter in the membrane was chosen with the use of the »Orientations of Proteins in membrane« (OPM) server [39]. The membrane consisted of 1-palmitoyl-2-oleoyl-sn-glycero-3-phosphocholine (POPC) and cholesterol in a molar ratio of 3:1. The system was electo-neutralized in 150 mM NaCl, and the TIP3P [40] water model was used. We constructed simulation systems, which approximated various steps in the alternating access cycle of the cotransporter. Therefore, we created IF systems in both the apo- conformation and the conformation with bound galactose, along with two sodium ions. The same procedure was followed for the OF system. In addition, for comparative purposes and to validate the system, we created an OF system with the bound inhibitor phlorizin.

The final systems consisted of approximately 94.000 atoms. The initial equilibration was conducted with the standard six-step procedure outlined by CHARMM-GUI, which consists of two steps of the NVT ensemble (the constant number of particles, constant volume, and temperature) followed by four steps of the NPT ensemble (the constant number of particles, constant pressure, and temperature) equilibration. The timestep used in the first three steps was 1 fs, while the last three steps of the equilibration scheme used the 2 fs timestep. Restraints to certain components in the system were relaxed during the subsequent stages. The equilibration procedure also consisted of 3000 steps of energy minimization using the steepest descent and the adaptive basis Newton–Raphson method.

Each system was exposed to a long equilibration phase of 100 ns. All simulations were carried out on GPUs with the CUDA version of the NAMD [41], version 2.13, molecular dynamics suite. The CHARMM36 [42] force field was used, which included parameters for galactose. Parameters for phlorizin were calculated using the ParamChem [43] server, and charges were refined with Gaussian and incorporated in the force field. All production simulations used the NPT ensemble and their lengths were at least 0.5 μs; some had a length of 1 μs, and all of the simulated systems also had replica simulations made. Temperature was held constant (303.15 K) using the Langevin thermostat with a dampening constant of 1 ps${}^{-1}$. The pressure was also held constant (1.0 bar) by the use of the Nose–Hover Langevin piston for pressure control [44,45]. The timestep of the production simulations was 2 fs and the SHAKE [46] algorithm was used. The cutoff for nonbonded interactions was set to 12 Å, and electrostatic interactions were calculated using the particle mesh Ewald method (PME).

### 4.4. Permeation Events and Water Permeability

Counting the water permeation events involves detecting all water molecules that permeate from the intracellular side into the extracellular side, and vice versa during the simulation. In order to exclude crossings due to PBC-wrapping, we define two planes parallel to the membrane, separated by Δ = 25 Å and positioned symmetrically, one on each side of the membrane’s mirror plane. In addition, we introduce two external slabs of thickness δ = 5 Å on both sides, indicating inner and outer compartments. If $z_{i}\left(t^{*}\right)$ is a *z* component of the CoM of the *i*-th water molecule and t* is the simulation time at which the molecule is found in the inner compartment fulfilling a condition $-\Delta /2-\delta <z_{i}\left(t*\right)<-\Delta /2$, then the molecule successfully permeates through the protein if it is detected in the outer compartment at a later time τ, such that $\Delta /2<z_{i}\left(t^{*}+\tau \right)<\Delta /2+\delta$. The same principle applies to the reverse direction. Furthermore, if the *z* component of the *i*-th water molecule is continuously confined within an interval −Δ/2,Δ/2 during t* and t*+τ, i.e., $-\Delta /2<z_{i}\left(t\in \left[t*,t*+\tau \right]\right)<\Delta /2$, then τ is exactly the time the *i*-th molecule needs to permeate a distance Δ through the protein.

The key characteristic quantities accounting for transport through the water channel are osmotic and diffusion permeabilities, $p_{f}$ and $p_{d}$ [15,47]. $p_{f}$ relates the net water flux $\Phi_{w}$ and impermeable solute concentration difference Δc at both channel ends, $\Phi_{w}=p_{f}\Delta c$. Providing the $\Phi_{w}$ can be written as a function of the water’s average drift velocity *v*, $\Phi_{w}=Sc_{w}v$, where $c_{w}$ is the water concentration, *S* is a channel cross-section, and *v* is further related to the frictional force $F_{\gamma }=\gamma v$, which equals the acting force on an individual water molecule due to osmotic pressure $\Pi =N_{A}k_{B}T\Delta c$; we arrive at the expression:

(1) $$\Phi_{w}=S\frac{k_{B}T}{\gamma L}\Delta c=p_{f}\Delta c,$$

where *L* is the channel length. Similar arguments can be used for relating the diffusive tracer flux to the difference between the inner and outer tracer concentrations. Therefore, assuming the inverse relationship between the molecular friction coefficient γ and diffusion, both permeabilities can be put into quantitative relationships with the channel opening *S* and a generalized diffusion constant *D*:

(2) $$p_{f/d}=S\times \left(\frac{D}{L}\right).$$

### 4.5. Intrinsic Protein Dynamics and Its Softness (Flexibility)

Crucial features underlying protein functions are encoded in intrinsic protein dynamics, which refer to collective thermal fluctuating motions in the configuration space defined by the protein structure. The ideal tool for identifying the collective motions of proteins is the principal component analysis (PCA) [48]. The first step in the PCA is to create a variance–covariance matrix, usually from the MD simulation data of a biomolecular system, where we remove rigid body translation,

(3) $$C_{ij}=\langle \left(r_{i}-\langle r_{i}\rangle \right)\left(r_{j}-\langle r_{j}\rangle \right)\rangle ,$$

where 〈〉 denotes the ensemble average (time average in the case of MD) and $r_{i}$ is an instantaneous position coordinate of the *i*-th atom or, alternatively, a center of mass of the selected group of atoms. C is a symmetric matrix that can be diagonalized by solving the secular equation

(4) $$\det\left(C-\lambda I\right)=0\;\equiv \;E^{T}CE=\lambda ,$$

where λ is a diagonal matrix, $\Delta_{ij}\lambda_{i}$, with eigenvalue $\lambda_{i}$, and $E=(e_{1},e_{2},\cdots )$ is the associated eigenvector matrix. Individual $\lambda_{i}$ can be expressed as the mean square of the collective mode coordinate fluctuations along the vector $e_{i}$. Thermal fluctuations can be well approximated by a multivariate normal distribution, which can be decomposed into the Boltzmann factors of individual harmonic vibrations. At thermal equilibrium, the energy is uniformly distributed over all available degrees of freedom. The same amount of thermal energy $k_{B}T/2$ populates each mode. Now, the premise is that an object fluctuates normally around its average position with a mean square amplitude $\langle \Delta x^{2}\rangle$ at temperature *T*. This type of motion can correspond to the vibration of an object on a harmonic spring with an effective force constant $k_{\mathrm{eff}}=k_{B}T/\Delta x^{2}$ [49], and can be applied to each collective mode obtained by the PCA. We define the mode-effective force constant, $\kappa_{i}=k_{B}T/\lambda_{i}$, which we may also call mode stiffness. Alternatively, we can introduce mode softness or mode flexibility, as reciprocal mode stiffnesses, $\sigma_{i}=\kappa_{i}^{-1}=\lambda_{i}/\left(k_{B}T\right)$, which is also proportional to the amplitude of the mode fluctuations. Flexibility is meaningful only for vibrational modes. However, particularly for the low-dimensional systems, some mixing between rotational and vibrational modes might persist due to the incomplete removal of the pure rigid rotation from the bead trajectory. We introduce an additional weighting factor $w_{vi}$, indicting the pure vibrational nature of the *i*-th mode, which can readily be calculated from the total kinetic energy of the mode, given that eigenvector components describe the bead displacements in a unit of time,

(5) $$w_{vi}=\frac{W_{tot}^{i}-W_{R}^{i}}{W_{tot}^{i}},\;W_{R}^{i}=\frac{{\Gamma_{i}}^{2}}{2J}=\frac{1}{2J}\sum_{k}{\left(m_{k}r_{ik}\times e_{ik}\right)}^{2},\;W_{tot}^{i}=\frac{1}{2}\sum_{k}m_{k}e_{ik}^{2},$$

where *k* runs over the beads. Now, the overall protein softness σ is defined by the sum over all PCA modes while taking into account only vibrational modes as follows:

(6) $$\sigma =\sum_{i}\sigma_{i}=\frac{1}{k_{B}T}\sum_{i}\lambda_{i}w_{vi}.$$

In this picture, higher flexibility indicates the presence of modes with more enhanced thermal fluctuations.

It is generally accepted that low-frequency modes are functionally relevant, but that does not imply that the lowest frequency mode corresponds exactly to the functional mode. The latter is more likely to emerge as a superposition of several PCA modes. Thus, looking for the presence of a specific functional mode, and making the comparison between different systems more consistent, we choose to predefine bead displacements in the form of a normalized (projecting) vector P with the same dimensions as eigenvectors e. If P encodes a specific type of motion, then the intensity of this mode is

(7) $$\sigma_{P}=\sum_{i}\sigma_{i}=\frac{1}{k_{B}T}\sum_{i}\lambda_{i}w_{vi}\left(P\cdot e_{i}\right).$$

In that respect, it might be convenient to project PCA modes along predetermined reference modes mimicking specific types of motion, for example, the rocking-bundle type.

If the PCA is performed on the mass-weighted coordinates, $q_{j}=\sqrt{m_{j}}r_{j}$,

(8) $${\tilde{C}}_{ij}=\langle \left(q_{i}-\langle q_{i}\rangle \right)\left(q_{j}-\langle q_{j}\rangle \right)\rangle ,\;{\tilde{E}}^{T}\tilde{C}\tilde{E}=\tilde{\lambda },$$

one obtains quasi-harmonic modes $\tilde{e_{i}}$ in $\tilde{E}$ and the corresponding vibrational frequencies, $\omega_{i}=\sqrt{k_{B}T/\tilde{\lambda_{i}}}$.

## 5. Conclusions

Our study suggests that the water transport in SGLT1, although in principle a passive transport, is strongly supported by the domain motion of the protein, which resembles the so-called “rocking bundle motion”. Along with the instantaneous channel opening, it is demonstrated that thermal fluctuations of domain motion play a role in modulating water permeation on a 100 ns timescale. This type of motion regulates transport in all conformational states, including outward-facing and inward-facing, as well as in complexes with the substrate and inhibitor.

The design of optimal inhibitors for water transport in SGLT1 should, thus, focus on identifying those with the greatest potential to enhance the stiffening effect on the rocking bundle-type dynamics, in addition to the closing effect on the water channel.

## Figures and Tables

**Table 1:** Total number of water permeation events, and diffusion and osmotic permeabilities of the s-OF and p-IF systems calculated for two 300 ns sections.

System	$N_{w}$	$p_{d}\times 10^{-15}$ [cm${}^{3}$/s]	$p_{f}\times 10^{-15}$ [cm${}^{3}$/s]	$p_{f}/p_{d}$
s-OF 100-400 ns	79	3.97	6.23	1.567
s-OF 400-700 ns	165	8.30	13.01	1.568
p-IF 100-400 ns	40	2.01	2.69	1.337
p-IF 400-700 ns	46	2.31	2.80	1.276

## Data Availability

All data are available upon reasonable request.

## Abbreviations

The following abbreviations are used in this manuscript:

SGLT1	human sodium–glucose cotransporter protein
MD	molecular dynamics
PCA	principle component analysis
